# 

**Published:** 1906-08

**Authors:** 


// THE AMERICAN
### Journal of Dental Science

Founded, Owned, Edited and Published by the Dental Profession of America.

THIRD SERIES.

VOLUME XXXVII. AUGUST, 1906. NO. 8.

EDITORS:
WM. GIRD BEECROFT, D. D. S., Madison, Wis. B. H. TEAGUE, D. D. S., Aiken, S. C
Published on the 10th day of each month.
Sub-cription Price, $1 00 per year. Foreign countries, $1.50 per year.
*Entered as Second-Class Matter, Madison, Wis.*
Address all communications, both business and editorial, to
The Dental Publishing Company, Madison, Wisconsin.

